# Supplementary material for: From sequence to enzyme mechanism using multi-label machine learning
Source: BMC Bioinformatics. 2014 May 19;15:150. doi: 10.1186/1471-2105-15-150 (PMC4229970; doi:10.1186/1471-2105-15-150)
Supplement: Additional file 2 — Java code of ml2db. Additional file ml2db_code.tar.gz contains the Java source code to run the multi-label machine learning experiments and save the results to database. The code’s Javadoc is included. [file 1471-2105-15-150-S2.zip › additional file 2/ml2db/ml2db/doc/mulan/classifier/meta/thresholding/MetaLabeler.html]

MetaLabeler


JavaScript is disabled on your browser.


- Overview
- Package
- Class
- Use
- Tree
- Deprecated
- Index
- Help

- Prev Class
- Next Class

- Frames
- No Frames

- All Classes

- Summary:
- Nested |
- Field |
- Constr |
- Method

- Detail:
- Field |
- Constr |
- Method


mulan.classifier.meta.thresholding

## Class MetaLabeler

- java.lang.Object
- - mulan.classifier.MultiLabelLearnerBase
  - - mulan.classifier.meta.MultiLabelMetaLearner
    - - mulan.classifier.meta.thresholding.Meta
      - - mulan.classifier.meta.thresholding.MetaLabeler

- All Implemented Interfaces:
  :   java.io.Serializable, MultiLabelLearner, weka.core.TechnicalInformationHandler

  ---

    

  ```
  public class MetaLabeler
  extends Meta
  ```

  Class implementing the MetaLabeler algorithm. For more information, see

  Version:
  :   2010.12.14

  Author:
  :   Marios Ioannou, George Sakkas, Grigorios Tsoumakas

  See Also:
  :   Serialized Form

- - ### Constructor Summary

    Constructors

    Constructor and Description

    `MetaLabeler()`

    Default constructor

    `MetaLabeler(MultiLabelLearner baseLearner,
    weka.classifiers.Classifier classifier,
    java.lang.String metaDataChoice,
    java.lang.String aClassChoice)`

    Constructor that initializes the learner
  - ### Method Summary

    Methods

    | Modifier and Type | Method and Description |
    | --- | --- |
    | `weka.core.TechnicalInformation` | `getTechnicalInformation()` Returns an instance of a TechnicalInformation object, containing detailed information about the technical background of this class, e.g., paper reference or book this class is based on. |
    | `java.lang.String` | `globalInfo()` Returns a string describing the multi-label learner. |
    | `void` | `setFolds(int f)` Sets the number of folds for internal cv |

    - ### Methods inherited from class mulan.classifier.meta.thresholding.Meta

      `getClassifier`
    - ### Methods inherited from class mulan.classifier.meta.MultiLabelMetaLearner

      `getBaseLearner`
    - ### Methods inherited from class mulan.classifier.MultiLabelLearnerBase

      `build, getDebug, isUpdatable, makeCopy, makePrediction, setDebug`
    - ### Methods inherited from class java.lang.Object

      `equals, getClass, hashCode, notify, notifyAll, toString, wait, wait, wait`

- - ### Constructor Detail


    - #### MetaLabeler

      ```
      public MetaLabeler()
      ```

      Default constructor


    - #### MetaLabeler

      ```
      public MetaLabeler(MultiLabelLearner baseLearner,
                 weka.classifiers.Classifier classifier,
                 java.lang.String metaDataChoice,
                 java.lang.String aClassChoice)
      ```

      Constructor that initializes the learner

      Parameters:
      :   `baseLearner` - the underlying multi-label learner
      :   `classifier` - the binary classification
      :   `metaDataChoice` - the type of meta-data
      :   `aClassChoice` - the type of the class
  - ### Method Detail


    - #### getTechnicalInformation

      ```
      public weka.core.TechnicalInformation getTechnicalInformation()
      ```

      **Description copied from class: `MultiLabelLearnerBase`**

      Returns an instance of a TechnicalInformation object, containing detailed
      information about the technical background of this class, e.g., paper
      reference or book this class is based on.

      **Specified by:**
      :   `getTechnicalInformation` in interface `weka.core.TechnicalInformationHandler`

      **Specified by:**
      :   `getTechnicalInformation` in class `MultiLabelLearnerBase`

      Returns:
      :   the technical information about this class


    - #### setFolds

      ```
      public void setFolds(int f)
      ```

      Sets the number of folds for internal cv

      Parameters:
      :   `f` - the number of folds


    - #### globalInfo

      ```
      public java.lang.String globalInfo()
      ```

      **Description copied from class: `MultiLabelLearnerBase`**

      Returns a string describing the multi-label learner.

      **Specified by:**
      :   `globalInfo` in class `MultiLabelLearnerBase`

      Returns:
      :   a description suitable for displaying in a future gui


- Overview
- Package
- Class
- Use
- Tree
- Deprecated
- Index
- Help

- Prev Class
- Next Class

- Frames
- No Frames

- All Classes

- Summary:
- Nested |
- Field |
- Constr |
- Method

- Detail:
- Field |
- Constr |
- Method
